# Left ventricular noncompaction highlighted by three-dimensional and speckle tracking echocardiography

**DOI:** 10.31744/einstein_journal/2022AI6853

**Published:** 2022-08-10

**Authors:** Viviane Tiemi Hotta, Luiz Mário Baptista Martinelli, Valdir Ambrósio Moisés, Charles Mady, Fábio Fernandes

**Affiliations:** 1 Hospital das Clínicas Faculdade de Medicina Universidade de São Paulo São Paulo SP Brazil Instituto do Coração (InCor), Hospital das Clínicas, Faculdade de Medicina, Universidade de São Paulo, São Paulo, SP, Brazil.; 2 Fleury Medicina e Saúde Grupo Fleury São Paulo SP Brazil Fleury Medicina e Saúde, Grupo Fleury, São Paulo, SP, Brazil.; 3 Escola Paulista de Medicina Universidade Federal de São Paulo São Paulo SP Brazil Escola Paulista de Medicina, Universidade Federal de São Paulo, São Paulo, SP, Brazil.

Left ventricular noncompaction (LVNC) is a relatively new and heterogeneous cardiomyopathy, first reported by Chin et al. in 1990.^(1)^ It is characterized by prominent ventricular trabeculations, intratrabecular recesses and a bi-layered myocardium, composed of compacted and noncompacted layers.^(1,2)^ Left ventricular noncompaction may be an isolated finding or associated to other cardiomyopathies, metabolic disorders and congenital heart diseases.^(2)^ It is believed to result from failure of ventricular compaction in the embryogenic phase, with increasing evidence of a genetic component.^(2)^

Since its first description, much has been learned about this disease, but there are some limitations for its diagnosis. Cardiac magnetic resonance imaging is considered the gold standard for diagnosis of LVNC, but echocardiography remains the first line imaging modality due to its availability and cost-effectiveness.

**Figure 1:**
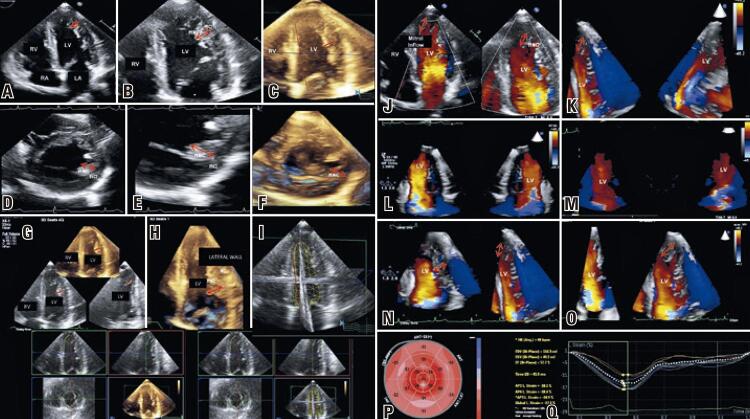
Images from conventional, three-dimensional and speckle tracking echocardiography. 2D echo and 3D echo, respectively, on apical 4-chamber view (A, B and C) and transversal view (D, E and F) depicting left ventricular noncompaction (red arrows). 3D echo on apical 4-chamber and transversal view of the same patient (C and F). 3D and triplanar views of the left ventricle (G, H and I). 2D (J) and 3D color Doppler (K to O) showing blood flow within the left ventricle recesses. Strain analysis with global longitudinal strain slightly decreased = -17% (P and Q)

Source: Hotta VT, Martinelli LM, Fernandes F, Moises VA, Vieira ML, Mady C. P697 left ventricular non compaction highlighted by three-dimensional and speckle tracking echocardiography [Poster Session i394]. Eur Heart J Cardiovasc Imaging. 2020;21(Supp 1):jez319.371.^(3)^

LA: left atrium; LV: left ventricle; RA: right atrium; RV: right ventricle; MNC: noncompacted myocardium; MC: compacted myocardium.

We report a case of an asymptomatic 21-year-old male, with no personal or family history of cardiomyopathy. Two-dimensional echocardiography (2D echo) showed increased left ventricular trabeculation in the apical segments of lateral and anterior walls, and a noncompacted myocardium/compacted + noncompacted myocardium ratio of 0.33, compatible with LVNC, according to Chin´s criteria.^(1)^ 3D echo provided more detailed left ventricle morphology analysis and 3D echo color Doppler demonstrated ventricular flow within the intraventricular recesses. Strain analysis by speckle tracking echocardiography (STE) evidenced global longitudinal strain = -17% (normal values <-18%), probably related to an incipient systolic dysfunction not shown by evaluation of left ventricular ejection fraction in 2D echo (Figure 1).

This case illustrates echocardiographic modalities for LVNC diagnosis. 3D echo and STE are technologies that may play an incremental role in evaluation of LVNC but need further investigation and validation.^(3)^
